# Determinants of Fertility Desire among Women Living with HIV in the Childbearing Age Attending Antiretroviral Therapy Clinic at Jimma University Medical Center, Southwest Ethiopia: A Facility-Based Case-Control Study

**DOI:** 10.1155/2020/6504567

**Published:** 2020-08-12

**Authors:** Nigusie Shifera Aylie, Lelisa Sena Dadi, Eshetu Alemayehu, Mengistu Ayenew Mekonn

**Affiliations:** ^1^Nursing Department, College of Health Science Mizan-Tepi Unversity, Mizan-Aman, Ethiopia; ^2^Epidemiology Department, Faculty of Public Health, Jimma University, Jimma, Ethiopia; ^3^Epidemiology Department, College of Health Science Mizan-Tepi Unversity, Mizan-Aman, Ethiopia

## Abstract

**Background:**

High fertility and aspiration to have more children are a normal phenomenon in many developing countries including Ethiopia. The desire of people living with HIV/AIDS (PLWHA) to have children can have significant public health implications. Despite the growing number of women living with HIV/AIDS, the issues of fertility and childbearing intention have not been widely studied in Ethiopia.

**Objective:**

To identify determinants of fertility desire among women living with HIV in the childbearing age attending antiretroviral therapy clinic at Jimma University Medical Center, Southwest Ethiopia.

**Methods:**

A facility-based case-control study was conducted in March 2019. Cases were women living with HIV who had fertility desire, and controls were those who had not. Data was collected using a face-to-face interview using a pretested questionnaire. The data was entered into EpiData 3.1 and exported to SPSS Version 24 for analysis. Bivariate and multivariable logistic regression analyses were used to identify candidate and independent determinants of fertility desire, respectively. Independent determinants associated with fertility desire were assessed using AORs with their corresponding 95% CIs at *P* value < 0.05 cutoff point*. Results*. Three hundred forty-four (115 cases and 229 controls) were included into the study with a 100% response rate. Age categories 15-24 (AOR: 4.1; 95% CI: 2.0, 8.4) and 25-34 (AOR: 2.3; 95% CI: 1.3, 4.2) years, not using family planning (AOR: 2.3; 95% CI: 1.4, 4.0), and having a sexual partner (AOR: 1.9; 95% CI: 1.1, 3.2) were independent predictors of fertility desire.

**Conclusions:**

Age of women, family planning, and sexual partner were found to be the independent predictors of fertility desire among women living with HIV/AIDS. Policymakers and health care providers who are working on an ART clinic should try to consider the effects of these factors for women living with HIV while developing HIV/AIDS interventions and discussing on sexual and reproductive health issues with their clients, respectively.

## 1. Introduction

Globally, an estimated 36.7 million people were living with HIV/AIDS; over 25.5 million of them are in Africa where 76% of all HIV-positive women live in sub-Saharan Africa. AIDS-related illnesses remain the leading cause of death among women of reproductive age (15–49 years). It is the second leading cause of death among young women aged 15–24 years globally, and the first in Africa [1]. In Ethiopia, HIV prevalence among women of the reproductive age (15–49 years) was 0.9% and the most affected group are those who are sexually active and economically productive falling within the 25–49 age group [2].

Women's desires for fertility can put them on danger against the preventive strategies for vertical transmission of HIV/AIDS. Worldwide, over 90% of HIV infections among young children are due to mother-to-child transmission (MTCT). In the absence of any appropriate intervention, transmission of HIV/AIDS ranges from 15% to 45%. This transmission rate can be reduced to under 5% with effective interventions during the times of pregnancy, labor, delivery, and breastfeeding [3].

The UNICEF report estimated that MTCT of HIV was 9% in Latin America and Caribbean, 15% in Eastern Europe and Central Asia, 16% in Middle East and Northern Africa, 18% in West and Central Africa, and 8% in East and Southern Africa [4]. In Ethiopia, a finding from systematic review showed that the pooled prevalence of MTCT of HIV was 9.93%. Furthermore, the study also showed that almost one in every ten HIV-exposed infants became HIV positive [5]. Moreover, Ethiopia was characterized by low PMTCT service coverage. For example, Ethiopian public health institute (EPHI) reports indicated that more than 30,818 (8%) mothers need PMTCT of HIV/AIDS that cannot be addressed in 2018 [6].

Evidence indicates that women living with HIV continue with desire of having more children at varying degrees. For example, two studies conducted in Canada [6] and Malawi [7] showed that the proportions of HIV-positive women who wanted to have children in the future were 69% and 17% [7, 8], respectively. In Ethiopia, different studies indicated different levels of fertility desire among HIV-positive women. A study done in Nekemt Town [9], Tigray, [10] and Addis Ababa [11] demonstrated that the proportions of women living with HIV who had fertility desire were 42.1%, 45.5%, and 56.2%, respectively.

Studies revealed that age [7, 10, 12–20], ethnicity [7, 10, 13], education status [9, 15, 18, 21], religion [19, 21], residence [7], marital status [10, 20–22], wealth status [18], abortion [13], children [12, 13, 20], parity [7, 8, 12, 20, 23], sexual violence [24], sexual activity [24], child HIV status [10], sexual partner [19, 23], partner desire [9, 13, 15, 17, 25], discussion [10], family planning [8, 21], family influence [20], CD4 count [10, 23], viral load [13], ART duration [10], HIV diagnosis, HIV disclosure [12], partner HIV status [9, 11], health status [22, 23], knowledge [15], effects of HIV on fertility [17], and perceived stigma [14] were among the factors associated with fertility desire.

In Ethiopia, limited studies have pointed out different factors that determine fertility desire of HIV-positive women [9, 10, 12, 20, 22]. However, no study has been conducted before in our study area to identify fertility desire of women living with HIV. Moreover, most of the previous studies does not assess HIV pregnancy-related knowledge and household wealth status, so this study strengthens the previous findings by addressing the above gaps.

The main aim of this study was to identify determinants of fertility desire among women living with HIV in Jimma University Medical Center, Southwest Ethiopia. So, this study had an implication for PMTCT of HIV/AIDS, the need for counseling to facilitate informed decision-making about childbearing, and the future demand for services for children born to infected women by identifying factors that determine fertility desire of women.

## 2. Methods

### 2.1. Study Setting, Design, and Period

An institution-based case-control study was conducted at Jimma University Medical Center (JUMC) in March 2019. Geographically, JUMC is located in Jimma City, which is 352 km to southwest of Addis Ababa. Currently, it is the only teaching and referral hospital in the southwestern part of the country, providing services to approximately 15,000 inpatient, 160,000 outpatient attendants, 11,000 emergency cases, and 4500 deliveries in a year. A total of 3221 HIV-positive individuals were on ART follow-up at JUMC ART clinic, of whom 1956 were females and 1775 were women in the reproductive age. Every stable client attends clinics every month, but clients with severe illness have frequent appointments within a month. The clinic provides counseling, tracing, ART treatment, and nutritional and medical treatment services.

### 2.2. Eligibility Criteria

All consecutively selected women living with HIV in the reproductive age (15–49) who had a follow-up in the ART clinics of Jimma University Medical Center were included in this study. Women who were pregnant, have severe mental illness, had hearing difficulties, and/or with confirmed infecundity were excluded.

### 2.3. Sample Size Determination and Sampling Technique

Sample size was estimated using two population proportion formulas for unmatched case-control study using Epi Info version 7 StatCalc function considering assumptions of 95% CI, 80% power, and a case-control ratio of 1 : 2. From a similar study conducted in Addis Ababa, health status of the women living with HIV was taken as the main predictor of outcome fertility desire. *P*_1_ representing the proportion of cases who are exposed to very good current health status was 60%, and *P*_2_ representing the proportion of controls who are exposed to very good current health status was 44.4% [11]. Then, after considering a 10% nonresponse rate, the total sample size was estimated to be 344 (cases = 115, controls = 229). Study participants were grouped as cases if they had fertility desire and controls if they did not have fertility desire among women living with HIV on ART follow-up. The actual participants were selected through a consecutive sampling technique based on the criteria of the case definition.

### 2.4. Measures and Operational Definitions

#### 2.4.1. Fertility Desires

It is a psychological state in which someone has the personal motivation to have a child. Those who have motivation to have more children in the future have fertility desire, and those who did not have motivation to have more children were considered not to have fertility desire during the study period [26].

#### 2.4.2. Sexually Active

Women who had at least one sexual practice during the last six months before the interview were labeled as sexually active [27].

#### 2.4.3. Good Knowledge

Those respondents who answered correctly at least 60% of the total knowledge questions are considered to have good knowledge [28].

#### 2.4.4. Poor Knowledge

Those respondents who correctly answered less than 60% of the total knowledge's questions are considered to have poor knowledge [28].

#### 2.4.5. Housewife

A housewife is a woman whose work is running or managing her family and does not have any other job [26].

#### 2.4.6. Wealth Index

Households are given scores based on the number and kinds of household assets, and the scores are derived using principal component analysis (PCA) [2].

#### 2.4.7. Sexual Violence

Sexual violence is any sexual act, attempt to obtain a sexual act, unwanted sexual comments or advances, or acts to traffic or otherwise directed against a person's sexuality using coercion, by any person regardless of their relationship to the victim, in any setting throughout their life [29].

#### 2.4.8. Viral Load

The amount of HIV in a sample of blood. Viral load (VL) is reported as the number of HIV RNA copies per milliliter of blood. In this study, it has three categories. The first category is TND which means target not detected or the virus is not at a detectable level. The second and third categories are the detectable level which is <20 copies and ≥20 copies HIV RNA copies per milliliter of blood, respectively [30].

### 2.5. Data Collection Procedures and Tools

After confirming eligibility and obtaining written informed consent, consecutively selected participants were asked to respond to an interviewer-administered questionnaire which was prepared in the local (Amharic and Affan Oromo) languages. The questionnaire assessed sociodemographic and socioeconomic characteristics, fertility desire, reproductive characteristics, clinical characteristics, and HIV pregnancy-related knowledge. Questions were adapted from previously conducted similar studies [7–25]. Medical records of HIV-positive women were reviewed to confirm HIV status and other relevant medical history, including date of HIV diagnosis, recent CD4 count, ART status, date of ART start, and viral load.

### 2.6. Data Management and Analysis

Data completeness was checked manually, then entered into EpiData 3.1 and exported to SPSS Version 24 for analysis. Pretest of the questionnaire was conducted in Shenen Gibe Hospital, which had similar services to the study hospital. Training was given to the data collectors and supervisors on the objective of the study, maintenance of ethical standards, and methods of data collection; hence, the data collectors were familiarized with data collection tools with respect to the study with practical exercises. Data collectors were blinded about the outcome of the study so that they did not distinguish cases and controls.

The study participants were identified/classified after data collection based on unique identification codes. Data coding and cleaning were performed by cross-checking to printout data for possible errors. Missing values and outliers were checked before analysis by running descriptive analysis. The household wealth index was computed using the PCA method by considering locally available household assets which were dummy coded. Before running the PCA, the requirements of factor analysis were checked.

The descriptive statistics were presented in frequency tables and graphs. Normality assumption was checked for age. Multicollinearity between the independent variables was checked. The association between fertility desire and each covariate was assessed first by bivariate logistic regression to identify candidate variables for the final model. Variables with *P* value *<* 0.25 were taken into multiple logistic regression analysis to identify independent predictors of fertility desire.

The backward likelihood ratio with 0.1 probability removal was used to develop the model. Goodness of fit of the final model was checked using the Hosmer-Lemeshow test of goodness of fit considering good fit at *P* value *>* 0.05, omnibus likelihood test < 0.05, and model classification of accuracy was checked. Finally, independent predictors of fertility desire were declared at *P* value < 0.05 cutoff point, and strength of the association was assessed using AORs with their corresponding 95% CIs.

### 2.7. Ethical Statement

The study was approved by the Research and Ethics Committee, of Jimma University Institute of Health. Then, relevant offices were communicated for their cooperation with a formal letter issued by the Research and Ethics Committee. The purpose of the study was verified briefly to the study participant, and confidentiality was assured. Finally, written consent was obtained from study participants before conducting the interview. For women, who were married and less than 18 years of age, in addition to the assent, permission of their husbands was obtained to include them in the study. But for study participants, who are less than 18 years of age, were not married, and are living independently, as there is no one to sign on behalf of the parent or guardian, only assents from the participant were obtained.

## 3. Results

### 3.1. Sociodemographic and Economic Characteristics

A total of 115 cases and 229 controls were included in the study with a 100% response rate. Three-fourth of the 87 (75.7%) cases and majority 185 (80.8%) of the controls were urban residents. Concerning the age of the study participants, the mean age of cases was 28.5 ± 5.6 SD and the mean age of controls was 32.7 ± 6.5 SD. Closely half 54 (47.0%) of the cases and 101 (44.1%) of the controls were Orthodox religion followers. Concerning marital status of the study participants, 53 (46.1%) of the cases and nearly half 111 (48.5%) of the controls were married.

In bivariate analysis, from all sociodemographic and economic characteristics, age of the respondents and household wealth status were potential candidates for multivariable logistic regression (see Table 1).

### 3.2. Reproductive Characteristics

**Figure 1:**
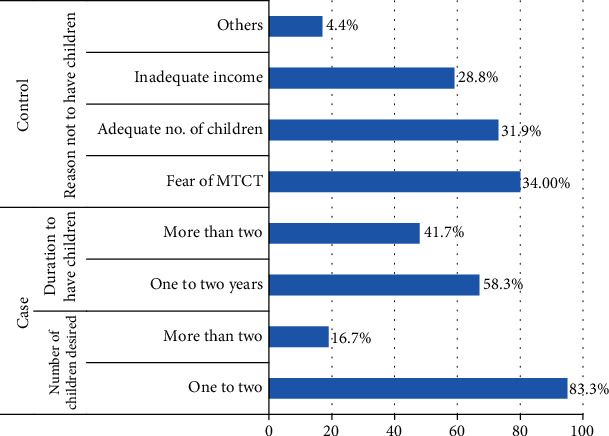
Fertility desire, number, and duration to have children among cases and reasons not to have children among controls of women living with HIV attending ART in Jimma University Medical Center, 2019 (*N* = 344).

Majority of the cases 95 (83.3%) reported to have planned to have one or two children where more than half 67 (58.3%) of the cases wished to have children within a two-year duration. Among controls, the main reason not to have children for about 80 (34.9%) were due to fear of mother-to-child transmission of HIV (see Figure 1).

Almost all 113 (98.2%) of the cases and 227 (99.1%) of the controls reported to have had sexual intercourse in their lifetime. About three-fourth 86 (76.1%) of the cases and closely two-third 139 (61.2%) of the controls were sexually active in the last 12 months preceding the study. More than half 65 (57.5%) of the cases and three-fourth (77.1%) of the controls had children. Majority of 89 (78.8%) cases and 161 (70.9%) controls had no history of abortion. Regarding family planning option, slightly below three-fourth of 82 (72.6%) cases had not used family planning and 126 (55.5%) controls used family planning. About two-third 75 (65.2%) of the cases and slightly above three-fourth 177 (77.3%) of the controls had no any history of sexual violence.

In bivariate analysis, from all reproductive characteristics, sexual activity in the last 12 months, having a sexual partner currently, lack of child, history of abortion, family planning, family support, family pressure to have children, and history of sexual violence were potential candidates for multiple logistic regression (see Table 2).

### 3.3. Pregnancy-Related Knowledge among Women on Follow-Up of ART

This study also assessed pregnancy-related knowledge of women attending ART. All cases and controls mentioned at least one way of HIV transmission and prevention. Almost all respondent 111 (96.5%) of the cases and 213 (93.0%) of the controls mentioned unsafe sex as a mode of HIV transmission. About 91 (80.5%) cases and 179 (78.9%) controls mentioned that HIV can be transmitted through breastfeeding. More than majority 89 (96.7%) of the cases and 197 (97.0%) of the controls mentioned through ART medication (see Table 3).

### 3.4. Clinical Characteristics

Majority of the cases 102 (88.7%) and controls 195 (85.2%) were tested for HIV before a year of the study. Almost a similar proportion of cases 102 (88.7%) and controls 194 (84.7%) were on ART for more than one year. Concerning their CD4 count, more than three-fourth of 90 (78.3%) cases and 158 (69.0%) controls had CD4 ≥ 350. Majority of 78 (67.8%) cases and half of 131 (51.7%) controls have their viral load cannot be detected (TND). About half 59 (51.3%) of the cases had poor knowledge whereas closely two-third 148 (64.6%) of the controls had good knowledge of HIV transmission and prevention.

In bivariate analysis from all clinical characteristics, CD4 count, viral load, and knowledge were potential candidates for multiple logistic regression (see Table 4).

### 3.5. Determinants of Fertility Desire

From the total variable entered in the multivariable regression, three variables were found to be independently associated with fertility desire among women living with HIV (see Table 5). The odds of fertility desire among the age group 15-24 years and 25-34 were four times (AOR: 4.1; 95% CI: 2.0, 8.4) and two times (AOR: 2.3; 95% CI: 1.3, 4.2) higher as compared to age range from 35 to 49 years, respectively.

The odds of fertility desire among women who had not used family planning were two times (AOR: 2.3; 95% CI: 1.4, 4.0) higher as compared to those women living with HIV who use family planning. The odds of fertility desire among women who had sexual partners were about two times (AOR: 1.9; 95% CI: 1.1-3.2) higher when compared to those women living with HIV who had no sexual partners (see Table 5).

## 4. Discussion

Understanding the desire for fertility among women living with HIV/AIDS has a significant role to reduce MTCT of HIV with the introduction of ART, which changes the views of childbearing despite having the disease [31].

Age of the women was significantly associated with fertility desire. Women of younger age had higher odds fertility desire as compared to older women. This finding is similar to that of studies from Brazil, Canada, USA, Guinea, Ghana, and Rwanda which indicate that young women living with HIV experience significantly more fertility desire than HIV-infected, older women [7, 13, 15–17, 25]. In Ethiopia, some studies demonstrated the presence of an association between age and fertility desire [10, 12, 20]. The possible similarity is that it is quite normal to have a fertility desire during this period b/c it is the peak of reproduction. In many societies, women are active in producing their offsprings during this period [32]. On the contrary in another study, being young was reported to be inversely associated with fertility desire [33]. The possible reason for the inconsistency may be sociocultural or methodological differences between study participants.

In the current study, the odds of fertility desire among women, who had not used family planning, were higher when compared with those who used family planning. This finding is supported by a study done in Uganda and Guinea [25, 34], which revealed that women who have not used any family planning are more likely to have fertility desire than those who used. A study in Ethiopia has reported the same finding [35]. This might be due to the fact that they have not attained their desired family size and unmet need of family planning service [36]. In contrast, having no family planning is negatively associated with fertility desire [37]. The possible difference might be due to poor economic status having less fertility desire. The health workers providing their chronic AIDS care must be made aware of this, and a range of contraceptive options should be made available, as well as all the associated information about these options.

Women who had sexual partners had higher odds of fertility desire as compared to women who had no sexual partners. This result is also in line with the study done in Tanzania and Guinea [23, 25] which stated that odds of fertility desire were high on women who had sexual partners than their counterparts. A study in Ethiopia reported the same finding [20]. Probably, this may be due to the fact that having sexual partners may play a role in preconception planning behaviors. Moreover, having male partners minimizes her economic, social, and psychological burden of HIV/AIDS [12]. Thus, policymakers and health care providers should consider the role of male partners while developing policy and running an HIV/AIDS intervention.

### 4.1. Study Limitation

Men were not included in the study, in spite of the essential role they can play in deciding about their family size. This study is also limited to women who are receiving chronic, long-term care for their HIV infection, despite fertility desire differing from women who are not receiving the care. Furthermore, this study included secondary school girls; most of them are not sexually active and may not want to have children shortly, and this may bias the findings of this study.

### 4.2. Conclusions and Recommendation

This study revealed that age of the women, family planning, and permanent sexual partner were the independent determinants of fertility desire among women living with HIV attending ART at JUMC. This finding has an implication for the health care provider and policymakers to consider the effects of these variables while discussing the reproductive option, family planning service, and safer conception; providing adequate information about PMTCT of HIV; and assisting them in making informed reproductive decision to minimize the risk of MTCT of HIV and unplanned pregnancy. Further studies including men and qualitative methods can help deepen understanding of fertility desire among women living with HIV on ART follow-up. Moreover, previous studies revealed that age is a known confounder of fertility desire, so we have recommended further studies with age-matched case-control analysis to control the effects of age.

## Figures and Tables

**Table 1:** Bivariate analysis of sociodemographic and economic characteristics of women living with HIV attending ART at Jimma University Medical Center, 2019 (*N* = 344).

Variables	Category	Cases (*n* = 115) No. (%)	Controls (*n* = 229) No. (%)	COR (95% CI)	*P* values
Age	15-24	38 (33.1)	31 (13.5)	5.9 (3.0-11.4)	0.001^∗^
	25-34	55 (47.8)	92 (40.2)	2.9 (1.6-5.1)	0.001^∗^
	35-49	22 (19.1)	106 (46.3)	1	
Residence	Urban	87 (75.7)	185 (80.8)	1	0.27
	Rural	28 (24.3)	44 (19.2)	0.7 (0.4-1.3)	
Religion	Orthodox	54 (47.0)	101 (44.1)	1	
	Muslim	36 (31.3)	77 (33.6)	0.9 (0.5-1.5)	0.610
	Protestant	21 (18.2)	44 (19.2)	0.9 (0.5-1.6)	0.718
	Others	4 (3.5)	7 (3.1)	1.1 (0.3-3.1)	0.918
Ethnicity	Oromo	56 (48.7)	116 (50.7)	1	
	Amhara	34 (29.6)	56 (24.4)	1.2 (0.7-2.1)	0.399
	Keffa	12 (10.4)	33 (14.4)	0.7 (0.4-1.6)	0.449
	Others	13 (11.3)	24 (10.5)	1.1 (0.5-2.4)	0.762
Marital status	Single	28 (24.3)	45 (19.7)	1	
	Married	53 (46.1)	111 (48.5)	0.8 (0.4-1.4)	0.366
	Divorced	11 (9.6)	25 (10.9)	0.7 (0.3-1.6)	0.425
	Widowed	11 (9.6)	23 (10.0)	0.7 (0.3-1.5)	0.381
	Separated	12 (10.4)	25 (10.9)	0.9 (0.3-2.5)	0.866
Educational level	Cannot read & write	9 (7.8)	19 (8.3)	1.3 (0.5-3.3)	0.542
	Read & write	25 (21.7)	54 (23.6)	1.4 (0.7-2.6)	0.362
	Elementary	28 (24.4)	61 (26.6)	1.3 (0.7-2.6)	0.335
	Secondary	24 (20.9)	49 (21.4)	1.4 (0.6-2.5)	0.463
	Tertiary	29 (25.2)	46 (20.1)	1	
Occupation	Housewife	30 (26.0)	53 (23.1)	1	
	Unemployed	10 (8.7)	28 (12.2)	0.6 (0.3-1.5)	0.288
	Daily labor	13 (11.3)	34 (14.8)	0.7 (0.3-1.5)	0.324
	Merchant	17 (14.8)	43 (13.8)	0.7 (0.3-1.4)	0.327
	Govt. employee	31 (27.0)	39 (17.0)	1.4 (0.7-2.7)	0.306
	Private business	14 (12.2)	32 (14.1)	0.8 (0.4-1.8)	0.513
Household wealth index	Poor	43 (37.4)	71 (31.0)	1	
	Medium	33 (28.6)	84 (36.7)	0.6 (0.4-1.1)	0.125^∗^
	Good	39 (33.9)	74 (32.3)	0.9 (0.5-1.5)	0.615

Key: ∗ = *P* value *<* 0.25; 1 = reference category.

**Table 2:** Bivariate analysis reproductive characteristics of women living with HIV attending ART in Jimma University Medical Center, 2019 (*N* = 344).

Variables	Category	Cases (*n* = 115) No. (%)	Controls (*n* = 229) No. (%)	COR (95% CI)	*P* values
Sexually active	Yes	86 (76.1)	139 (61.2)	2.0 (1.2-3.3)	0.007^∗^
	No	27 (24.0)	88 (38.8)	1	
Sexual partner	Yes	81 (71.7)	110 (48.5)	2.7 (1.7-4.4)	0.001^∗^
	No	32 (28.3)	117 (51.5)		
HIV disclosure	Yes	63 (77.8)	83 (75.5)	1.1 (0.6-2.2)	0.709
	No	18 (22.2)	27 (24.5)	1	
Partner fertility desire	Yes	57 (70.4)	77 (70.0)	1.0 (0.5-1.9)	0.956
	No	24 (29.6)	33 (30.0)	1	
Children	Yes	65 (57.5)	175 (77.1)	1	
	No	48 (42.5)	52 (22.9)	2.5 (1.5-4.0)	0.001^∗^
History of abortion	Yes	24 (21.2)	66 (29.1)	1	
	No	89 (78.8)	161 (70.9)	1.5 (0.9-2.6)	0.124^∗^
Family planning	Yes	31 (27.4)	126 (55.5)	1	
	No	82 (72.6)	101 (44.5)	3.3 (2.0-5.4)	0.001^∗^
Family support	Yes	82 (71.3)	126 (55.0)	2.0 (1.3-3.3)	0.004^∗^
	No	33 (28.7)	103 (45.0)	1	
Pressure from family to have children	Yes	57 (49.6)	77 (33.6)	1.9 (1.2-3.0)	0.004^∗^
	No	58 (50.4)	152 (66.4)	1	
Discussion with heath professional	Yes	83 (72.2)	161 (70.3)	1.1 (0.7-1.8)	0.719
	No	32 (27.8)	68 (29.7)	1	
History of sexual violence	Yes	40 (34.8)	52 (22.7)	1.8 (1.1-3.0)	0.018^∗^
	No	75 (65.2)	173 (73.3)	1	

Key: ∗ = *P* value *<* 0.25; 1 = reference category.

**Table 3:** Assessments of HIV pregnancy-related knowledge among women living with HIV attending ART at Jimma University Medical Center, 2019 (*N* = 344).

Variables	Category		Cases (*n* = 115) No. (%)	Cases (*n* = 115) No. (%)
HIV transmission	Common use of sharp material	Yes	108 (93.4)	211 (92.1)
		No	7 (6.1)	18 (7.9)
	Unsafe sex	Yes	111 (96.5)	213 (93.0)
		No	4 (3.5)	16 (7.0)
	Blood transfusion	Yes	35 (30.4)	56 (24.5)
		No	80 (69.6)	173 (75.5)
HIV prevention	Faithful	Yes	62 (53.9)	147 (64.2)
		No	53 (46.1)	82 (36.0)
	Abstinence	Yes	91 (79.1)	182 (79.5)
		No	24 (20.9)	47 (20.6)
	Safe sexual practice	Yes	73 (63.5)	143 (63.2)
		No	42 (36.5)	126 (36.8)
MTCT of HIV	During pregnancy	Yes	72 (63.7)	158 (69.6)
		No	41 (36.3)	69 (30.4)
	Labor and delivery	Yes	64 (56.6)	145 (63.9)
		No	49 (43.4)	82 (36.1)
	Breastfeeding	Yes	91 (80.5)	179 (78.9)
		No	22 (19.5)	48 (21.1)
PMTCT of HIV	ART medication	Yes	89 (96.7)	197 (97.0)
		No	3 (3.3)	6 (3.0)
	Condom use	Yes	20 (22.0)	23 (11.4)
		No	71 (78.0)	179 (88.9)
Infant feeding option	Infant formula, no breast milk	Yes	23 (24.0)	63 (29.9)
		No	73 (76.0)	148 (70.1)
	Cow's milk, no breast milk	Yes	16 (16.7)	38 (18.0)
		No	80 (83.3)	173 (82.0)
	Breast milk only for six months	Yes	74 (77.1)	170 (80.6)
		No	22 (22.9)	41 (19.4)

**Table 4:** Bivariate analysis of clinical characteristics of women living with HIV attending ART in Jimma University Medical Center, 2019 (*N* = 344).

Variables	Category	Cases (*n* = 115) No. (%)	Controls (*n* = 229) No. (%)	COR (95% CI)	*P* values
Tested time of HIV	<1 year	13 (11.3)	34 (14.8)	0.7 (0.4-1.4)	0.368
	≥1year	102 (88.7)	195 (85.7)	1	
Started time of ART	<1 year	13 (11.3)	35 (15.3)	0.7 (0.3-1.4)	0.317
	≥1 year	102 (88.7)	194 (84.7)	1	
CD4 count	<350	25 (21.7)	71 (31.0)	1	
	≥350	90 (78.3)	158 (69.0)	1.6 (0.9-2.7)	0.072^∗^
Viral load	TND	78 (67.8)	131 (57.2)	1.6 (0.9-3.1)	0.129^∗^
	<20	21 (18.3)	54 (23.6)	1.1 (0.5-2.3)	0.863
	>20	16 (13.9)	44 (19.2)	1	
Partner tested for HIV	Yes	64 (79.0)	81 (73.6)	1	
	No	17 (21)	29 (25.4)	0.7 (0.4-1.5)	0.391
Partner HIV status	Positive	47 (73.6)	54 (66.7)	1	
	Negative	17 (26.4)	27 (33.3)	0.7 (0.3-1.5)	0.379
Health status	Improving	105 (91.3)	202 (88.2)	1	
	Same	10 (8.7)	27 (11.8)	0.7 (0.3-1.5)	0.384
Discrimination by health professionals	Yes	12 (10.4)	19 (8.3)	1.2 (0.6-2.7)	0.514
	No	103 (89.6)	210 (91.7)	1	
Knowledge	Poor	59 (51.3)	81 (35.4)	1.9 (1.2-3.0)	0.005^∗^
	Good	56 (48.7)	148 (64.6)	1	

Key: ∗ = *P* value *<* 0.25; 1 = reference category; TND = target not detected.

**Table 5:** Multivariable analysis of factors associated with fertility desire among women living with HIV attending ART in Jimma University Medical Center, 2019 (*N* = 344).

Variables	Category	Cases (*n* = 115) No. (%)	Controls (*n* = 229) No. (%)	COR (95% CI)	AOR (95% CI)	*P* values
Age	15-24	38 (33.1)	31 (13.5)	5.9 (3.0-11.4)	4.1 (2.0-8.4)	<0.001^∗∗^
	25-34	55 (47.8)	92 (40.2)	2.9 (1.6-5.1)	2.3 (1.3-4.2)	0.005^∗∗^
	35-49	22 (19.1)	106 (46.3)	1	1	
Household wealth index	Poor	43 (37.4)	71 (31.0)	1	1	
	Medium	33 (28.6)	84 (36.7)	0.6 (0.4-1.1)	0.7 (0.4-1.2)	0.206
	Good	39 (33.9)	74 (32.3)	0.9 (0.5-1.5)	0.9 (0.5-1.6)	0.715
History of sexual violence	Yes	40 (34.8)	52 (22.7)	1.8 (1.1-3.0)	0.9 (0.4-1.6)	0.764
	No	75 (65.2)	173 (73.3)	1	1	
Family support	Yes	82 (71.3)	126 (55.0)	2.0 (1.3-3.3)	1.0 (0.5-2.0)	0.913
	No	33 (28.7)	103 (45.0)	1	1	
Pressure from family to have children	Yes	57 (49.6)	77 (33.6)	1.9 (1.2-3.0)	0.8 (0.4-1.6)	0.588
	No	58 (50.4)	152 (66.4)	1	1	
Sexually active	Yes	86 (76.0)	139 (61.2)	2.0 (1.2-3.3)	0.8 (0.3-1.9)	0.629
	No	27 (24.0)	88 (38.8)	1	1	
Sexual partner currently	Yes	81 (71.7)	110 (48.5)	2.7 (1.7-4.4)	1.9 (1.1-3.2)	0.015^∗∗^
	No	32 (28.3)	117 (51.5)	1	1	
Viral load	TND	78 (67.8)	131 (57.2)	1.6 (0.9-3.1)	1.3 (0.6-2.7)	0.498
	<20	21 (18.3)	54 (23.6)	1.1 (0.5-2.3)	1.1 (0.4-2.5)	0.875
	>20	16 (13.9)	44 (19.2)	1	1	
CD4 count	<350	25 (21.7)	71 (31.0)	1	1	
	≥350	90 (78.3)	158 (69.0)	1.6 (0.9-2.7)	1.6 (0.9-2.9)	0.093
Children	Yes	65 (57.5)	175 (77.1)	1	1	
	No	48 (42.5)	52 (22.9)	2.5 (1.5-4.0)	0.7 (0.3-1.3)	0.237
Family planning	Yes	31 (27.4)	126 (55.5)	1	1	
	No	82 (72.6)	101 (44.5)	3.3 (2.0-5.4)	2.3 (1.4-4.0)	0.001^∗∗^
History of abortion	Yes	24 (21.2)	66 (29.1)	1	1	
	No	89 (78.8)	161 (70.9)	1.5 (0.9-2.6)	0.7 (0.4-1.4)	0.433
HIV pregnancy-related knowledge	Poor	59 (51.3)	81 (35.4)	1.9 (1.2-3.0)	0.8 (0.4-1.5)	0.521
	Good	56 (48.7)	148 (64.6)	1	1	

Key: ∗∗ = *P* value < 0.05; 1 = reference category; TND = target not detected.

## Data Availability

The dataset generated and analyzed during the study will be made available to organizations and individuals based on reasonable request.

## Abbreviations

AIDS: Acquired immune deficiency syndrome
AOR: Adjusted odds ratio
ART: Antiretroviral therapy
COR: Crude odds ratio
EDHS: Ethiopia Demographic and Health Survey
HIV: Human immunodeficiency virus
JUMC: Jimma University Medical Center
MTCT: Mother-to-child transmission
PLWHA: People living with HIV/AIDS
PMTCT: Preventing mother-to-child transmission
TND: Target not detected
UNAID: United Nations Program on HIV/AIDS
WHO: World Health Organization.
